# MicroRNAs mediate liver transcriptome changes upon soy diet intervention in mice

**DOI:** 10.1111/jcmm.14140

**Published:** 2019-01-08

**Authors:** Edward Seclaman, Loredana Balacescu, Ovidiu Balacescu, Cristina Bejinar, Mihai Udrescu, Catalin Marian, Ioan Ovidiu Sirbu, Andrei Anghel

**Affiliations:** ^1^ Department of Biochemistry and Pharmacology University of Medicine and Pharmacy “Victor Babes” Timisoara Timisoara Romania; ^2^ Department of Functional Genomics, Proteomics and Experimental Pathology The Oncology Institute "Prof. Dr. Ion Chiricuta" Cluj‐Napoca Romania; ^3^ Department of Computer and Information Technology Politehnica University of Timisoara Timisoara Romania

## Abstract

Soy‐based diets have triggered the interest of the research community due to their beneficial effects on a wide variety of pathologies like breast and prostate cancer, diabetes and atherosclerosis. However, the molecular details underlying these effects are far from being completely understood; several recent attempts have been made to elucidate the soy‐induced liver transcriptome changes in different animal models. Here we used Next Generation Sequencing to identify a set of microRNAs specifically modulated by short‐term soy‐enriched diet in young male mice and estimated their impact on the liver transcriptome assessed by microarray. Clustering and topological community detection (CTCD) network analysis of STRING generated interactions of transcriptome data led to the identification of four topological communities of genes characteristically altered and putatively targeted by microRNAs upon soy diet intervention.

## 1 INTRODUCTION

Soy‐based/rich diets have been associated with decreased risk of breast and prostate cancer, type 2 diabetes and atherosclerotic pathologies, an effect attributed to soy proteins and/or associated isoflavones.^1^ Several transcriptomic studies have investigated the effect of soy components on vertebrate physiology and metabolism, emphasizing the central role of the liver in mediating these effects.^2^, ^3^, ^4^ However, nothing is known about the impact of soy diet on liver microRNA expression and their role in mediating the soy‐associated transcriptome changes. In the present study, we used the Next Generation Sequencing (NGS) and microarray to assess the expression of microRNAs and mRNAs in the liver of adult male mice fed for 4 weeks a soy‐rich diet. By combining miRWalk3.0 prediction and STRING algorithms with a clustering and topological community detection (CTCD) approach, we analysed and characterized the functional gene communities impacted by microRNAs upon soy diet intervention.

## 2 METHODS

### 2.1 Dietary intervention

Two groups of three 12 weeks old male mice housed in Udel^®^ polysulphone cages, on a 12 hour light‐dark cycle were fed ad libitum granulated regular chow (Cantacusino Institute, Bucharest) and granulated soy‐enriched chow (25% soy bean) for 28 days. On day 28, the animals were sacrificed and approximately 0.5 g of liver tissues have been collected, immediately, immersed in RNAlater stabilization solution (Qiagen) and stored at −80°C until its further use.

### 2.2 Small RNA sequencing

Small RNA was isolated using the miRNeasy Mini Kit (Qiagen); RNA concentration was determined using the Qubit 2.0 Fluorometer (Thermo Fisher Scientific) and RNA quality control was assessed using an Agilent 2100 Bioanalyzer (median RIN value of 8.8). Small RNA sequencing was performed on a NextSeq 500 Sequencer (Illumina) platform (Biogazelle, Belgium).

Mapped data were filtered using a cut‐off of four reads, normalized using the DESeq2 geometric mean‐based method, log2‐transformed, followed by calculation of differential miRNA expression using a False Discovery Rate (FDR) of 5% (Benjamini & Hochberg). qRT‐PCR validation was performed on RNA samples purified with miRVANA kit (Thermofisher) using Taqman assays (Thermofisher) and normalization to U6 snRNA (Thermofisher). RNA‐Seq data have been deposited at NCBI Gene Expression Omnibus under accession code GSE113598.

### 2.3 Microarray expression profiling and qPCR

Total RNA containing miRNA was extracted with TriReagent (Sigma‐Aldrich), purified with RNeasy Mini Kit (Qiagen) and assessed for quality (RIN > 8) with Agilent 2100 Bioanalyzer (Agilent Technologies).

The cRNA‐Cy3 microarray probes synthesized from 100 ng of total RNA (Agilent Low Input Quick Amp Labeling Kit) were hybridized on Agilent G3 Mouse Gene Expression v.2 arrays for 17 hours at 65°C. After washing, the hybridized slides were scanned with an Agilent G2505C Microarray Scanner at 3 µm resolution (protocol GE1_1105_Oct12), the microarray images were processed with Agilent Feature Extraction (FE) software v. 11.5.1.1 and data analysis was done in R/Bioconductor (https://www.bioconductor.org/) using as input the raw median signals generated by FE. The differential expression was assessed using linear models and empirical Bayes statistics implemented in limma package/R^5^ and p‐values adjusted for multiple testing with Benjamini‐Hochberg method. The microarray data have been deposited at NCBI Gene Expression Omnibus under accession code GSE111804.

qRT‐PCR validation of seven of the differentially expressed genes (Cyp4a14, Tle1, Ugdh, Fh1, Esr1, Hamp2, Cebpe) was performed on RNA samples purified with miRVANA kit (Thermofisher) using Taqman assays (Thermofisher) and normalization to Gapdh and Hprt (Thermofisher).

### 2.4 Bioinformatics analysis

Target prediction for the differentially expressed microRNAs was computed using the miRWalk3.0 platform (adjusted binding probability > 0.95).^6^

The functional interactions between the genes found deregulated upon soy‐diet intervention were retrieved using the STRING platform (https://string-db.org//; medium confidence interaction score) and further analysed using a CTCD approach which assumes both modularity‐class and force directed layout clustering, as previously described.^7^, ^8^, ^9^, ^10^ In our graph representation, each vertex represents a gene/protein and each edge stands for all types of gene interactions (ie either up‐ and down‐regulation) between two genes/proteins. Modularity classes are indicated by assigning a distinct colour to each community and associate with distinct biological functions.^11^

## 3 RESULTS

### 3.1 The soy‐enriched diet

Despite the biochemical changes triggered by soy addition (File S1; Supplementary Table S1), the overall change of the soy‐enriched diet energetic value is minimal (+6.1%), which is reflected by the lack of significant weight gain in the soy‐fed group compared to control (14.2% vs 6.8%, *P* = 0.224).

**Table 1:** MicroRNAs deregulated in the liver of soy‐fed animals

Gene	Coordinates	log_2 _FC	P adj
mmu‐miR‐145a‐5p	chr18: 61647825‐61647894 [−]	0.849	1.32E‐02
mmu‐miR‐455‐3p	chr4: 63256851‐63256932 [+]	1.201	1.5E‐02

FC: fold change.

### 3.2 microRNA expression

Soy‐fed vs control liver analysis generated an average of 22.3 × 10^6^ reads mapped to 439 mature microRNAs and 20.573 × 10^6^ reads mapped to 434 mature microRNAs, respectively; none of these differences are statistically significant.

Deseq analysis with Benjamini & Hochberg correction (FDR = 5%) identified two differentially expressed microRNAs (mm‐miR145a‐5p and mmu‐miR‐455‐3p, Table 1), both validated by qRT‐PCR analysis (FC = 1.21 and 1.56 respectively).

### 3.3 mRNA expression

Our microarray analysis of mRNAs expressed in the liver of soy‐fed animals identified a set of 202 differentially expressed unique genes (adjusted *P* value < 0.05), of which 68.32% are up‐regulated (FC > 1.5) and 31.68% are down‐regulated (FC < 0.66) (File S1; Supplementary Table S2). We have validated by Taqman qRT‐PCR seven genes: Cyp4A14 (FC = 146.06; *P* = 0.024), Ugdh (FC = 2.16; *P* = 0.0018), Tle1 (FC = 0.42, *P* = 0.00035), Fh1 (0.36; *P* = 0.0057), ESR1 (FC = 0.55; *P* = 0.0079), Hamp2 (FC = 0.012; *P* = 0.039), Cebpe (0.0087; *P* = 0.0007).

### 3.4 Network analysis

**Figure 1:**
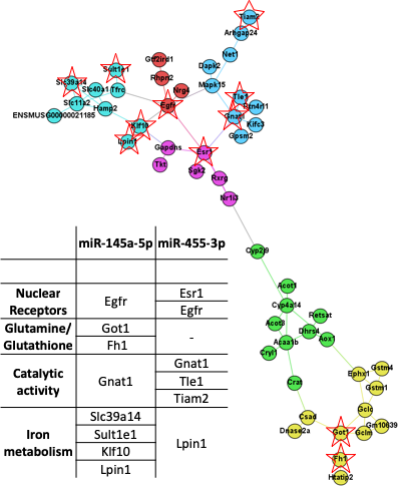
Network analysis: Analysis of liver microRNA‐mRNAs relationships upon soy diet intervention based on a clustering and topological community detection procedure applied to STRING data generated from the differential gene expression network. Vertex/gene positions are assigned by employing force‐directed network layout Force Atlas 2. The distinct colours indicate modularity classes associated with functional properties (Blue ‐ catalytic activity community, Turquoise ‐ Iron metabolism community, Red ‐ Egfr (nuclear receptor) community, Pink ‐ Esr1/RXR (nuclear receptor) community, Green ‐ Xenobiotic/fatty acid metabolizing community, Yellow ‐ Glutamine/Glutathione metabolizing community). mmu‐miR‐145a‐5p and mmu‐miR‐455‐3p miRWalk3.0 predicted targets are marked with a star and outlined in tabular form

In order to understand the biological significance of our transcriptome data, we used a CTCD approach to analyse the functional protein‐protein interactions network of the differentially expressed genes generated by STRING10.0 algorithms (http://string-db.org/). We identified five gene communities, partially overlapping with, but also complementing the STRING functional networks (File S1; Supplementary Table S3). Our CTCD network analysis delineates a central axis of transcription factors (Egfr, Esr1, RXRg, NR1i3), which links the iron metabolism community (centred on Slc40a1, Slc39a14, Slc11a2 and Tfrc), catalytic activity community (centered on Gnat, Tle1 and Tiam2), glutamate/glutathione metabolism community (centred on Gclm, Gclc, Got1, Gstm1, Gstm4), and xenobiotic/fatty acid metabolism community (centred on Cyp2j9, Cyp4A14, Acaa1b, Aox1 and Acot1, Acot3, Ces1d, Pdk4). Of the 50 clustered genes, only 11 are down‐regulated, and predicted (TarPmiR algorithm, binding probabilities > 0.95 and binding energies < −18 kcal/mol) to interact with miR‐145 and miR‐455: Esr1, Gapdhs, Egfr, Nrg4 (nuclear receptor cluster), Got1, Fh1 (glutamine/glutathione cluster), Gnat1, Tle1, Tiam2 (catalytic activity cluster), Klf10, Hamp2, Slc39a14, Sult1e1, Lpin1 (iron metabolism cluster) (Figure 1).

## 4 DISCUSSION

Our microarray analysis identified transcriptome changes suggestive for an alteration of the liver defense against xenobiotics: Cyp4a14 (female‐predominant cytochrome P450 monooxygenase involved in detoxification through oxidation) and Aox1 (involved in the first‐pass, non‐CYP metabolism of xenobiotics) are strongly up‐regulated, while Sult1a1 and Sult1e1 (3′‐phospho‐5′‐adenylyl sulphate‐dependent sulfotransferases) are strongly down‐regulated.^12^, ^13^

Furthermore, in concordance with previous results, our data show changes in the expression of genes involved in glutamine/glutathione metabolism.^2^ Both the catalytic (Gclc) and the modifier (Gclm) units of glutathione‐cysteine ligase, as well as glutathione S‐transferase (Gstm1) genes are coordinately up‐regulated upon soy diet intervention, portraying a liver ready to detoxify peroxidized lipids and/or xenobiotics.^14^

At least some of the soy‐induced liver transcriptome changes are mediated by changes in the expression of mmu‐miR‐145a‐5p and mmu‐miR‐455‐3p. Both miR‐145 and miR‐455 have recently emerged as powerful markers of liver pathology, ranging from fibrosis to hepatocellular carcinoma (HCC).^15^ MiR‐145 is involved in the outgrowth of the embryonic hepatic bud and modulates the activation of stellate cells.^16^, ^17^ The role of miR‐455 in liver biology is less clear; a recent paper documents its role in Runx2‐mediated modulation of HCC migration, data that correlate with a predicted role in regulating cell adhesion and locomotion during liver metastasis of colorectal cancer.^18^ Of note, none of the liver‐specific (miR16, miR‐27, miR‐30, miR‐126) or liver most abundant microRNAs (miR‐122, miR‐192, miR‐199a/b, miR‐101, miR‐99a, and let‐7a/b/c/f) are deregulated upon soy dietary intervention.^19^, ^20^, ^21^, ^22^

Our data strongly suggest that miR‐145 and miR‐455 may impact the expression of nuclear receptors (Esr1, Egfr, Nrg4) and the glutamine/glutathione (Got1, Fh1), catalytic activity (Gnat1, Tle1, Tiam2) and iron metabolism (Klf10, Hamp2, Slc39a14, Sult1e1, Lpin1) clusters.

The changes in expression levels of iron transporters are particularly interesting as both protein extracts and soy phytates chelate and inhibit iron absorption and bioavailability, reducing both ferritin and iron serum levels.^23^, ^24^ On the other hand, the (miR‐dependent) down‐regulation of Esr1 is in line with published data showing that soy isoflavones exert their estrogenic effect through preferential transactivation of ESR2, effect manifest especially in low endogenous oestrogen level conditions (men, menopause, post‐ovariectomy).^25^, ^26^ It is thus plausible that the oestrogenic effect of the soy‐enriched diet is due to soy isoflavones signalling through an unperturbed ESR2 which may regulate target genes expression in both liganded and unliganded form.^27^, ^28^

Several lines of evidence suggest a bi‐directional crosstalk between both ESR and EGFR signalling and the two differentially regulated microRNAs. Oestrogen inhibits miR‐145 expression in splenic lymphocytes and epithelial endometrial cells, while miR‐145 targets Esr1 in human breast cancer cells.^29^, ^30^, ^31^ Similarly, EGFR down‐regulates the expression of miR‐145 in lung cancer cells, while miR‐145 and miR‐455 down‐regulate EGFR expression in human lung adenocarcinoma and gastric cancer cells, respectively.^32^, ^33^, ^34^

In this context, one of the emerging concepts tempting to speculate upon is that of a bi‐univocal feedback loop between ESR‐EGFRs and miR145/miR‐455. It would be interesting to assess whether, in the context of soy dietary challenge, this phenomenon is specific to the liver and to decipher the interplay of ESR1, ESR2, EGFR and microRNAs in liver's transcriptome response.

Our data reveal microRNA and mRNA changes induced by the 4 weeks of soy diet in the liver of young male mice and suggest that the liver transcriptome response to soy diet (in particular the ESR/EGFR expression and the iron metabolism) is partially mediated by two microRNAs, mmu‐miR‐145a‐5p and mmu‐miR‐455‐3p. One should nevertheless interpret these data as the sum of changes in all the types of cell composing the liver: hepatocytes, stellate cells, Kupffer cells, endothelial cells, blood cells; in this respect, it would be interesting to dissect the exact contributions of these cells to the overall transcriptome change. Whether the microRNA response to soy diet is cell type specific and age and/or gender biased, remains to be established by future experiments.

## CONFLICT OF INTEREST

The authors declare no competing interests.

## AUTHORS’ CONTRIBUTION

Edward Seclaman analysed RNA‐seq and microarray data, and contributed to the manuscript Andrei Anghel analysed qPCR‐data and contributed to the manuscript, Cristina Bejinar purified RNA, performed qRT‐PCR experiments, Ovidiu Balacescu and Loredana Balacescu performed microarray experiments and analysed microarray data, Mihai Udrescu performed CTCD network analysis, Catalin Marian analysed data and contributed to the manuscript, Ioan Ovidiu Sirbu designed the experiments, analysed data and wrote the manuscript. All authors reviewed the manuscript.
