# Supplementary material for: MicroRNAs mediate liver transcriptome changes upon soy diet intervention in mice
Source: J Cell Mol Med. 2019 Jan 8;23(3):2263–7. doi: 10.1111/jcmm.14140 (PMC6378209; doi:10.1111/jcmm.14140)
Supplement: Supplementary file 1 [file JCMM-23-2263-s001.docx]

**Supplementary table S1.** Biochemical composition of regular and soy-enriched chow

| **Parameter** | **Regular food** | **Soy-enriched food** | **Variation (%)** |
| --- | --- | --- | --- |
| Total carbohydrates (g/100g) | 64.83 | 57.62 | -11.12 |
| Available carbohydrates (g/100g) | 49.3 | 39.9 | -19.07 |
| Total sugar (g/100g) | 3.69 | 5.19 | 40.65 |
| Total diet fibres (g/100g) | 15.53 | 17.63 | 13.52 |
|  |  |  |  |
| **Lipids (g/100g)** | 2.86 | 6.88 | 140.56 |
| Saturated fatty acids (g/100g) | 0.48 | 1.07 | 122.92 |
| Mono-unsaturated fatty acids (g/100g) | 0.9 | 1.62 | 80.00 |
| Poly-unsaturated fatty acids (g/100g) | 1.46 | 4.14 | 183.56 |
| Trans fatty acids (g/100g) | <0,10 | <0,10 | - |
| Omega3 fatty acids (g/100g) | 0.08 | 0.47 | 487.50 |
| Omega6 fatty acids (g/100g) | 1.38 | 3.67 | 165.94 |
| Omega9 fatty acids (g/100g) | 0.87 | 1.52 | 74.71 |
| Omega6/omega3 ratio | 17.25 | 7.81 | -54.73 |
|  |  |  |  |
| **Proteins (g/100g)** | 18.09 | 22.13 | 22.33 |
|  |  |  |  |
| **Energy (Kcal/100g)** | 326 | 346 | 6.13 |

**Supplementary table S2.** Genes differentially regulated in the liver of young male mice upon soy-diet intervention.

| **Gene Symbol** | **Gene Name** | **FC** | **P. Value** | **adj. P.Val** |
| --- | --- | --- | --- | --- |
| Cyp4a14 | cytochrome P450, family 4, subfamily a, polypeptide 14 | 9.72 | 1.21E-05 | 1.33E-02 |
| Btbd9 | BTB (POZ) domain containing 9 | 6.35 | 3.62E-09 | 5.78E-05 |
| Acot3 | acyl-CoA thioesterase 3 | 5.20 | 1.50E-04 | 3.94E-02 |
| Gm10639 | predicted gene 10639 | 4.65 | 8.46E-05 | 3.29E-02 |
| Mab21l3 | mab-21-like 3 (C. elegans) | 3.88 | 1.72E-06 | 5.48E-03 |
| Peg3 | paternally expressed 3 | 3.75 | 1.06E-06 | 4.21E-03 |
| Pdk4 | pyruvate dehydrogenase kinase, isoenzyme 4 | 3.66 | 9.37E-07 | 4.21E-03 |
| Hist1h1c | histone cluster 1, H1c | 3.64 | 2.38E-06 | 6.75E-03 |
| Acot1 | acyl-CoA thioesterase 1 | 3.40 | 2.37E-05 | 1.86E-02 |
| Fam89a | family with sequence similarity 89, member A | 3.35 | 7.69E-05 | 3.23E-02 |
| Gm10872 | predicted gene 10872 | 3.25 | 1.31E-05 | 1.33E-02 |
| Aox1 | aldehyde oxidase 1 | 3.23 | 1.28E-04 | 3.66E-02 |
| Kcnk1 | potassium channel, subfamily K, member 1 | 3.20 | 3.59E-07 | 2.36E-03 |
| Meig1 | meiosis expressed gene 1 | 3.03 | 4.41E-05 | 2.43E-02 |
| Slc35f2 | solute carrier family 35, member F2 | 2.97 | 5.43E-06 | 9.43E-03 |
| Abca8b | ATP-binding cassette, sub-family A (ABC1), member 8b | 2.91 | 2.32E-05 | 1.86E-02 |
| Spc25 | SPC25, NDC80 kinetochore complex component, homolog (S. cerevisiae) | 2.90 | 4.78E-06 | 9.43E-03 |
| Paqr7 | progestin and adipoQ receptor family member VII | 2.90 | 1.30E-05 | 1.33E-02 |
| Ces1d | carboxylesterase 1D | 2.82 | 2.84E-05 | 2.01E-02 |
| Rtn4rl1 | reticulon 4 receptor-like 1 | 2.80 | 1.02E-04 | 3.38E-02 |
| Hunk | hormonally upregulated Neu-associated kinase | 2.79 | 2.79E-05 | 2.01E-02 |
| Cldn1 | claudin 1 | 2.79 | 3.22E-05 | 2.17E-02 |
| Slc16a13 | solute carrier family 16 (monocarboxylic acid transporters), member 13 | 2.79 | 1.14E-05 | 1.29E-02 |
| Gm38426 | predicted gene, 38426 | 2.77 | 2.10E-04 | 4.53E-02 |
| Htatip2 | HIV-1 tat interactive protein 2, homolog (human) | 2.77 | 3.20E-06 | 7.67E-03 |
| Acot2 | acyl-CoA thioesterase 2 | 2.73 | 4.46E-05 | 2.43E-02 |
| Rxrg | retinoid X receptor gamma | 2.73 | 5.28E-06 | 9.43E-03 |
| A730009E18Rik | RIKEN cDNA A730009E18 gene | 2.72 | 5.89E-05 | 2.89E-02 |
| 5330421C15Rik | RIKEN cDNA 5330421C15 gene | 2.71 | 8.01E-06 | 1.09E-02 |
| Csad | cysteine sulfinic acid decarboxylase | 2.70 | 6.31E-06 | 9.43E-03 |
| Paqr7 | progestin and adipoQ receptor family member VII | 2.69 | 4.32E-05 | 2.43E-02 |
| Gclc | glutamate-cysteine ligase, catalytic subunit | 2.67 | 4.04E-05 | 2.40E-02 |
| Tjp3 | tight junction protein 3 | 2.62 | 2.32E-04 | 4.73E-02 |
| Retsat | retinol saturase (all trans retinol 13,14 reductase) | 2.61 | 1.03E-04 | 3.38E-02 |
| 9030617O03Rik | RIKEN cDNA 9030617O03 gene | 2.59 | 3.94E-07 | 2.36E-03 |
| Gm11967 | predicted gene 11967 | 2.53 | 8.74E-05 | 3.35E-02 |
| Lrrc14b | leucine rich repeat containing 14B | 2.50 | 1.20E-04 | 3.64E-02 |
| Pnkd | paroxysmal nonkinesiogenic dyskinesia | 2.42 | 1.55E-04 | 4.02E-02 |
| Crat | carnitine acetyltransferase | 2.38 | 3.48E-05 | 2.31E-02 |
| Ephx1 | epoxide hydrolase 1, microsomal | 2.38 | 3.13E-05 | 2.14E-02 |
| Phka2 | phosphorylase kinase alpha 2 | 2.32 | 4.86E-05 | 2.61E-02 |
| Gm30698 | predicted gene, 30698 | 2.32 | 4.96E-05 | 2.64E-02 |
| 4930415C11Rik | RIKEN cDNA 4930415C11 gene | 2.28 | 3.76E-06 | 8.58E-03 |
| Tfrc | transferrin receptor | 2.28 | 1.80E-05 | 1.57E-02 |
| Lgalsl | lectin, galactoside binding-like | 2.25 | 1.08E-04 | 3.46E-02 |
| Glo1 | glyoxalase 1 | 2.25 | 5.33E-06 | 9.43E-03 |
| Etnppl | ethanolamine phosphate phospholyase | 2.25 | 7.25E-05 | 3.17E-02 |
| Gstm4 | glutathione S-transferase, mu 4 | 2.24 | 3.21E-06 | 7.67E-03 |
| Sgk2 | serum/glucocorticoid regulated kinase 2 | 2.24 | 1.19E-04 | 3.61E-02 |
| Dnase2a | deoxyribonuclease II alpha | 2.23 | 4.21E-05 | 2.40E-02 |
| Entpd5 | ectonucleoside triphosphate diphosphohydrolase 5 | 2.23 | 6.85E-06 | 9.94E-03 |
| Acaa1b | acetyl-Coenzyme A acyltransferase 1B | 2.20 | 7.07E-06 | 9.94E-03 |
| Frat1 | frequently rearranged in advanced T cell lymphomas | 2.20 | 2.09E-04 | 4.53E-02 |
| Cryl1 | crystallin, lambda 1 | 2.19 | 1.25E-05 | 1.33E-02 |
| Ugdh | UDP-glucose dehydrogenase | 2.17 | 9.31E-05 | 3.37E-02 |
| Gm5065 | predicted gene 5065 | 2.16 | 1.97E-04 | 4.46E-02 |
| Atxn7l1 | ataxin 7-like 1 | 2.16 | 2.40E-06 | 6.75E-03 |
| Parp6 | poly (ADP-ribose) polymerase family, member 6 | 2.15 | 6.42E-05 | 2.93E-02 |
| Kifc3 | kinesin family member C3 | 2.15 | 1.00E-04 | 3.37E-02 |
| Asap3 | ArfGAP with SH3 domain, ankyrin repeat and PH domain 3 | 2.14 | 8.10E-05 | 3.28E-02 |
| Gstm1 | glutathione S-transferase, mu 1 | 2.14 | 6.02E-06 | 9.43E-03 |
| Slc11a2 | solute carrier family 11 (proton-coupled divalent metal ion transporters), member 2 | 2.11 | 1.16E-04 | 3.55E-02 |
| Pllp | plasma membrane proteolipid | 2.11 | 5.34E-05 | 2.72E-02 |
| Nrp2 | neuropilin 2 | 2.11 | 3.54E-05 | 2.32E-02 |
| Gadd45b | growth arrest and DNA-damage-inducible 45 beta | 2.10 | 1.59E-04 | 4.03E-02 |
| AI661453 | expressed sequence AI661453 | 2.08 | 8.12E-07 | 4.21E-03 |
| Net1 | neuroepithelial cell transforming gene 1 | 2.08 | 4.22E-05 | 2.40E-02 |
| Ppfibp2 | PTPRF interacting protein, binding protein 2 (liprin beta 2) | 2.08 | 2.86E-05 | 2.01E-02 |
| Rhpn2 | rhophilin, Rho GTPase binding protein 2 | 2.07 | 1.36E-05 | 1.34E-02 |
| D17H6S56E-5 | DNA segment, Chr 17, human D6S56E 5 | 2.06 | 1.72E-04 | 4.14E-02 |
| Mir99ahg | Mir99a and Mirlet7c-1 host gene (non-protein coding) | 2.05 | 1.79E-04 | 4.25E-02 |
| Keg1 | kidney expressed gene 1 | 2.04 | 9.77E-05 | 3.37E-02 |
| Leo1 | Leo1, Paf1/RNA polymerase II complex component, homolog (S. cerevisiae) | 2.04 | 2.38E-04 | 4.79E-02 |
| Hist1h1c | histone cluster 1, H1c | 2.00 | 4.08E-05 | 2.40E-02 |
| Gpsm2 | G-protein signalling modulator 2 (AGS3-like, C. elegans) | 1.98 | 1.05E-06 | 4.21E-03 |
| Gm7160 | predicted gene 7160 | 1.93 | 2.47E-04 | 4.89E-02 |
| Gm19696 | predicted gene, 19696 | 1.93 | 1.39E-04 | 3.81E-02 |
| Blvrb | biliverdin reductase B (flavin reductase (NADPH)) | 1.92 | 1.80E-05 | 1.57E-02 |
| Als2cl | ALS2 C-terminal like | 1.92 | 8.30E-05 | 3.28E-02 |
| Snx24 | sorting nexing 24 | 1.92 | 8.95E-06 | 1.16E-02 |
| Pvrl1 | poliovirus receptor-related 1 | 1.92 | 9.73E-06 | 1.20E-02 |
| Erc2 | ELKS/RAB6-interacting/CAST family member 2 | 1.89 | 1.33E-04 | 3.72E-02 |
| Mfap3l | microfibrillar-associated protein 3-like | 1.89 | 6.33E-05 | 2.93E-02 |
| Tbc1d2 | TBC1 domain family, member 2 | 1.89 | 7.48E-05 | 3.17E-02 |
| Adora1 | adenosine A1 receptor | 1.89 | 3.73E-05 | 2.38E-02 |
| Cyp2j9 | cytochrome P450, family 2, subfamily j, polypeptide 9 | 1.89 | 1.46E-04 | 3.88E-02 |
| 4930402H24Rik | RIKEN cDNA 4930402H24 gene | 1.86 | 1.98E-04 | 4.46E-02 |
| Arhgap24 | Rho GTPase activating protein 24 | 1.85 | 1.26E-04 | 3.66E-02 |
| Gm10030 | predicted gene 10030 | 1.85 | 2.58E-05 | 1.96E-02 |
| Mapk15 | mitogen-activated protein kinase 15 | 1.84 | 1.61E-04 | 4.03E-02 |
| Wdr91 | WD repeat domain 91 | 1.84 | 1.42E-04 | 3.81E-02 |
| Smim3 | small integral membrane protein 3 | 1.83 | 1.95E-04 | 4.46E-02 |
| Crtc3 | CREB regulated transcription coactivator 3 | 1.82 | 1.00E-04 | 3.37E-02 |
| H2-Ea-ps | histocompatibility 2, class II antigen E alpha, pseudogene | 1.81 | 8.06E-05 | 3.28E-02 |
| Srxn1 | sulfiredoxin 1 homolog (S. cerevisiae) | 1.81 | 1.89E-04 | 4.39E-02 |
| Nr1i3 | nuclear receptor subfamily 1, group I, member 3 | 1.80 | 1.76E-04 | 4.20E-02 |
| Slc48a1 | solute carrier family 48 (heme transporter), member 1 | 1.80 | 1.25E-04 | 3.66E-02 |
| Creg1 | cellular repressor of E1A-stimulated genes 1 | 1.80 | 5.64E-06 | 9.43E-03 |
| Rtp3 | receptor transporter protein 3 | 1.80 | 1.10E-04 | 3.50E-02 |
| Lsamp | limbic system-associated membrane protein | 1.80 | 2.42E-04 | 4.83E-02 |
| 1810034E14Rik | RIKEN cDNA 1810034E14 gene | 1.79 | 2.69E-05 | 2.01E-02 |
| Zfp318 | zinc finger protein 318 | 1.78 | 1.04E-05 | 1.21E-02 |
| Shroom3 | shroom family member 3 | 1.78 | 3.72E-05 | 2.38E-02 |
| Rabep1 | rabaptin, RAB GTPase binding effector protein 1 | 1.78 | 8.73E-06 | 1.16E-02 |
| Syt3 | synaptotagmin III | 1.75 | 5.91E-05 | 2.89E-02 |
| Vmn1r216 | vomeronasal 1 receptor 216 | 1.75 | 2.03E-05 | 1.73E-02 |
| A130022F02Rik | RIKEN cDNA A130022F02 gene | 1.74 | 7.07E-05 | 3.16E-02 |
| Lrrc20 | leucine rich repeat containing 20 | 1.74 | 5.02E-06 | 9.43E-03 |
| Slc40a1 | solute carrier family 40 (iron-regulated transporter), member 1 | 1.73 | 2.16E-04 | 4.61E-02 |
| Gtf2ird1 | general transcription factor II I repeat domain-containing 1 | 1.73 | 1.28E-04 | 3.66E-02 |
| Abcc3 | ATP-binding cassette, sub-family C (CFTR/MRP), member 3 | 1.71 | 2.13E-05 | 1.79E-02 |
| Tenm3 | teneurin transmembrane protein 3 | 1.70 | 7.49E-05 | 3.17E-02 |
| Bik | BCL2-interacting killer | 1.70 | 6.49E-05 | 2.93E-02 |
| Dapk2 | death-associated protein kinase 2 | 1.69 | 1.58E-04 | 4.03E-02 |
| Tkt | transketolase | 1.69 | 2.38E-05 | 1.86E-02 |
| Akr1c20 | aldo-keto reductase family 1, member C20 | 1.68 | 5.15E-05 | 2.70E-02 |
| Vmo1 | vitelline membrane outer layer 1 homolog (chicken) | 1.67 | 1.43E-05 | 1.37E-02 |
| Galk1 | galactokinase 1 | 1.67 | 8.24E-05 | 3.28E-02 |
| Abhd8 | abhydrolase domain containing 8 | 1.66 | 2.50E-04 | 4.90E-02 |
| Zfand5 | zinc finger, AN1-type domain 5 | 1.66 | 6.13E-05 | 2.93E-02 |
| Svil | supervillin | 1.66 | 1.98E-04 | 4.46E-02 |
| Rnf32 | ring finger protein 32 | 1.65 | 6.44E-05 | 2.93E-02 |
| Bahcc1 | BAH domain and coiled-coil containing 1 | 1.64 | 2.08E-04 | 4.53E-02 |
| Clstn3 | calsyntenin 3 | 1.63 | 2.45E-04 | 4.87E-02 |
| Prdm9 | PR domain containing 9 | 1.62 | 2.52E-04 | 4.90E-02 |
| Fancb | Fanconi anemia, complementation group B | 1.62 | 1.72E-04 | 4.14E-02 |
| Fam210a | family with sequence similarity 210, member A | 1.61 | 2.42E-04 | 4.83E-02 |
| Traf5 | TNF receptor-associated factor 5 | 1.59 | 2.09E-04 | 4.53E-02 |
| Spats2 | spermatogenesis associated, serine-rich 2 | 1.59 | 2.52E-04 | 4.90E-02 |
| Thy1 | thymus cell antigen 1, theta | 1.59 | 2.02E-04 | 4.50E-02 |
| Dhrs4 | dehydrogenase/reductase (SDR family) member 4 | 1.58 | 7.95E-05 | 3.28E-02 |
| Gclm | glutamate-cysteine ligase, modifier subunit | 1.57 | 1.13E-04 | 3.52E-02 |
| LOC105247651 | uncharacterized LOC105247651 | 1.54 | 9.18E-05 | 3.37E-02 |
| 1700052K11Rik | RIKEN cDNA 1700052K11 gene | 1.54 | 2.35E-04 | 4.77E-02 |
| Tfcp2l1 | transcription factor CP2-like 1 | 1.53 | 1.89E-04 | 4.39E-02 |
| Arpp19 | cAMP-regulated phosphoprotein 19 | 1.52 | 9.14E-05 | 3.37E-02 |
| Pcsk6 | proprotein convertase subtilisin/kexin type 6 | 1.51 | 1.69E-04 | 4.13E-02 |
| Cc2d1b | coiled-coil and C2 domain containing 1B | 1.51 | 1.28E-04 | 3.66E-02 |
| Llgl2 | lethal giant larvae homolog 2 (Drosophila) | 1.51 | 1.69E-04 | 4.13E-02 |
| Gm10369 | predicted gene 10369 | 1.50 | 1.01E-04 | 3.37E-02 |
| Gch1 | GTP cyclohydrolase 1 | 0.65 | 2.24E-04 | 4.66E-02 |
| 0610037L13Rik | RIKEN cDNA 0610037L13 gene | 0.64 | 1.88E-04 | 4.39E-02 |
| Fh1 | fumarate hydratase 1 | 0.63 | 1.05E-04 | 3.42E-02 |
| Marveld1 | MARVEL (membrane-associating) domain containing 1 | 0.62 | 1.91E-04 | 4.39E-02 |
| Hspb8 | heat shock protein 8 | 0.62 | 8.56E-05 | 3.30E-02 |
| Parp9 | poly (ADP-ribose) polymerase family, member 9 | 0.62 | 8.44E-05 | 3.29E-02 |
| Tle1 | transducin-like enhancer of split 1, homolog of Drosophila E(spl) | 0.61 | 1.47E-05 | 1.38E-02 |
| Mbd1 | methyl-CpG binding domain protein 1 | 0.61 | 9.65E-05 | 3.37E-02 |
| Hs6st1 | heparan sulfate 6-O-sulfotransferase 1 | 0.60 | 2.81E-05 | 2.01E-02 |
| Cers6 | ceramide synthase 6 | 0.59 | 1.59E-04 | 4.03E-02 |
| Gm16006 | predicted gene 16006 | 0.59 | 3.84E-05 | 2.40E-02 |
| Vgll4 | vestigial like 4 (Drosophila) | 0.59 | 9.17E-05 | 3.37E-02 |
| Golt1b | golgi transport 1 homolog B (S. cerevisiae) | 0.59 | 4.09E-05 | 2.40E-02 |
| Gm16548 | predicted gene 16548 | 0.58 | 2.96E-05 | 2.05E-02 |
| Gm11827 | predicted gene 11827 | 0.57 | 1.67E-04 | 4.13E-02 |
| Slc39a14 | solute carrier family 39 (zinc transporter), member 14 | 0.57 | 9.34E-05 | 3.37E-02 |
| D030063E12 | uncharacterized protein D030063E12 | 0.56 | 2.28E-04 | 4.69E-02 |
| Lifr | leukemia inhibitory factor receptor | 0.55 | 1.22E-04 | 3.64E-02 |
| Pear1 | platelet endothelial aggregation receptor 1 | 0.55 | 1.52E-04 | 3.96E-02 |
| Klf10 | Kruppel-like factor 10 | 0.55 | 1.12E-04 | 3.52E-02 |
| Hip1r | huntingtin interacting protein 1 related | 0.54 | 1.15E-04 | 3.55E-02 |
| Sbsn | suprabasin | 0.54 | 4.47E-05 | 2.43E-02 |
| Sult1a1 | sulfotransferase family 1A, phenol-preferring, member 1 | 0.53 | 2.22E-04 | 4.63E-02 |
| Creb3l2 | cAMP responsive element binding protein 3-like 2 | 0.53 | 4.15E-05 | 2.40E-02 |
| Ulk1 | unc-51 like kinase 1 | 0.52 | 3.88E-05 | 2.40E-02 |
| Atp4a | ATPase, H+/K+ exchanging, gastric, alpha polypeptide | 0.52 | 1.22E-04 | 3.64E-02 |
| Slc43a1 | solute carrier family 43, member 1 | 0.50 | 9.27E-05 | 3.37E-02 |
| Serpina3g | serine (or cysteine) peptidase inhibitor, clade A, member 3G | 0.49 | 9.98E-05 | 3.37E-02 |
| 1700001L05Rik | RIKEN cDNA 1700001L05 gene | 0.49 | 1.13E-04 | 3.52E-02 |
| Fam187b | family with sequence similarity 187, member B | 0.48 | 1.05E-04 | 3.42E-02 |
| Sult1e1 | sulfotransferase family 1E, member 1 | 0.47 | 2.00E-04 | 4.47E-02 |
| Gapdhs | glyceraldehyde-3-phosphate dehydrogenase, spermatogenic | 0.47 | 1.48E-04 | 3.92E-02 |
| 1700113H08Rik | RIKEN cDNA 1700113H08 gene | 0.46 | 2.21E-04 | 4.63E-02 |
| Aen | apoptosis enhancing nuclease | 0.45 | 2.49E-04 | 4.90E-02 |
| Egfr | epidermal growth factor receptor | 0.45 | 1.64E-06 | 5.48E-03 |
| Plac9a | placenta specific 9a | 0.44 | 3.99E-06 | 8.68E-03 |
| Slc37a1 | solute carrier family 37 (glycerol-3-phosphate transporter), member 1 | 0.44 | 5.83E-05 | 2.89E-02 |
| Slc25a25 | solute carrier family 25 (mitochondrial carrier, phosphate carrier), member 25 | 0.43 | 6.41E-05 | 2.93E-02 |
| Gm826 | predicted gene 826 | 0.43 | 2.22E-04 | 4.63E-02 |
| Cela2a | chymotrypsin-like elastase family, member 2A | 0.43 | 1.01E-04 | 3.37E-02 |
| Acmsd | amino carboxymuconate semialdehyde decarboxylase | 0.43 | 5.24E-05 | 2.70E-02 |
| 1810055G02Rik | RIKEN cDNA 1810055G02 gene | 0.41 | 4.20E-05 | 2.40E-02 |
| Irf5 | interferon regulatory factor 5 | 0.41 | 1.22E-04 | 3.64E-02 |
| Serpina12 | serine (or cysteine) peptidase inhibitor, clade A (alpha-1 antiproteinase, antitrypsin), member 12 | 0.40 | 1.39E-06 | 5.11E-03 |
| Aim1l | absent in melanoma 1-like | 0.40 | 1.41E-04 | 3.81E-02 |
| Pnpla5 | patatin-like phospholipase domain containing 5 | 0.36 | 1.27E-04 | 3.66E-02 |
| Arhgef26 | Rho guanine nucleotide exchange factor (GEF) 26 | 0.34 | 3.14E-06 | 7.67E-03 |
| Esr1 | estrogen receptor 1 (alpha) | 0.33 | 1.34E-04 | 3.72E-02 |
| Pfkfb3 | 6-phosphofructo-2-kinase/fructose-2,6-biphosphatase 3 | 0.31 | 1.49E-07 | 1.45E-03 |
| Fam134b | family with sequence similarity 134, member B | 0.31 | 1.09E-04 | 3.46E-02 |
| Mafb | v-maf musculoaponeurotic fibrosarcoma oncogene family, protein B (avian) | 0.30 | 1.40E-04 | 3.81E-02 |
| Gnat1 | guanine nucleotide binding protein, alpha transducing 1 | 0.30 | 6.50E-05 | 2.93E-02 |
| Gm38832 | predicted gene, 38832 | 0.30 | 2.29E-04 | 4.69E-02 |
| Nrg4 | neuregulin 4 | 0.25 | 1.98E-04 | 4.46E-02 |
| Gpcpd1 | glycerophosphocholine phosphodiesterase GDE1 homolog (S. cerevisiae) | 0.25 | 6.07E-06 | 9.43E-03 |
| Chrna4 | cholinergic receptor, nicotinic, alpha polypeptide 4 | 0.22 | 2.14E-04 | 4.58E-02 |
| Got1 | glutamic-oxaloacetic transaminase 1, soluble | 0.21 | 9.75E-06 | 1.20E-02 |
| Fabp5 | fatty acid binding protein 5, epidermal | 0.17 | 1.38E-04 | 3.81E-02 |
| Adgrf1 | adhesion G protein-coupled receptor F1 | 0.16 | 1.51E-07 | 1.45E-03 |
| Lpin1 | lipin 1 | 0.11 | 5.57E-05 | 2.81E-02 |
| Tiam2 | T cell lymphoma invasion and metastasis 2 | 0.10 | 1.22E-09 | 2.93E-05 |
| Cebpe | CCAAT/enhancer binding protein (C/EBP), epsilon | 0.04 | 2.15E-07 | 1.71E-03 |
| Hamp2 | hepcidin antimicrobial peptide 2 | 0.03 | 9.97E-05 | 3.37E-02 |

**Suppementary Table 3. String analysis of differentially expressed genes upon soy diet intervention.** The STRING-generated network is of rather low complexity (178 nodes; 83 edges; average node degree 0,933; average clustering coefficient 0,178; expected number of edges 40; PPI enrichment value 2,04e-09) outlining significant effects on sulfur compound, oxoacid and unsaturated fatty acids metabolic processes, glutathione and cysteine metabolism and on ferrous iron transmembrane transporter activity.

| **Biological function** | | | |
| --- | --- | --- | --- |
| **#pathway ID** | **pathway description** | **observed gene count** | **false discovery rate** |
| GO.0006790 | Sulfur compound metabolic process | 13 | 2.92E-04 |
| GO.0043436 | Oxoacid metabolic process | 19 | 4.43E-03 |
| GO.0044699 | Single-organism process | 103 | 4.43E-03 |
| GO.0044710 | Single-organism metabolic process | 49 | 4.43E-03 |
| GO.1903874 | Ferrous iron transmembrane transport | 3 | 4.43E-03 |
| GO.0019752 | carboxylic acid metabolic process | 18 | 4.76E-03 |
| GO.0044281 | Small molecule metabolic process | 26 | 5.38E-03 |
| GO.0044763 | Single-organism cellular process | 94 | 8.21E-03 |
| GO.0006749 | Glutathione metabolic process | 5 | 1.62E-02 |
| GO.0006629 | Lipid metabolic process | 19 | 2.65E-02 |
| GO.0060586 | Multicellular organismal iron ion homeostasis | 3 | 2.65E-02 |
| GO.0006534 | Cysteine metabolic process | 3 | 3.50E-02 |
| GO.0044272 | Sulfur compound biosynthetic process | 6 | 4.49E-02 |
| **Molecular Function (GO)** | | | |
| GO.0015093 | Ferrous iron transmembrane transporter activity | 3 | 7.67E-03 |
| GO.0048037 | Cofactor binding | 11 | 8.76E-03 |
| **KEGG Pathway** | | | |
| 1040 | Biosynthesis of unsaturated fatty acids | 4 | 5.80E-03 |
| 1100 | Metabolic pathways | 24 | 5.80E-03 |
